# Participation of ABCA1 Transporter in Pathogenesis of Chronic Obstructive Pulmonary Disease

**DOI:** 10.3390/ijms22073334

**Published:** 2021-03-24

**Authors:** Stanislav Kotlyarov

**Affiliations:** Department of Nursing, Ryazan State Medical University, 390026 Ryazan, Russia; SKMR1@yandex.ru

**Keywords:** chronic obstructive pulmonary disease, COPD, inflammation, ABCA1, reverse cholesterol transport, innate immune system

## Abstract

Chronic obstructive pulmonary disease (COPD) is the important medical and social problem. According to modern concepts, COPD is a chronic inflammatory disease, macrophages play a key role in its pathogenesis. Macrophages are heterogeneous in their functions, which is largely determined by their immunometabolic profile, as well as the features of lipid homeostasis, in which the ATP binding cassette transporter A1 (ABCA1) plays an essential role. The objective of this work is the analysis of the ABCA1 protein participation and the function of reverse cholesterol transport in the pathogenesis of COPD. The expression of the ABCA1 gene in lung tissues takes the second place after the liver, which indicates the important role of the carrier in lung function. The participation of the transporter in the development of COPD consists in provision of lipid metabolism, regulation of inflammation, phagocytosis, and apoptosis. Violation of the processes in which ABCA1 is involved may be a part of the pathophysiological mechanisms, leading to the formation of a heterogeneous clinical course of the disease.

## 1. Introduction

Chronic obstructive pulmonary disease (COPD) is one of the most widespread diseases, it has great medical significance due to the high frequency of temporary and persistent disability and mortality. Social and economic burden of the disease is becoming more evident for health care systems as well as patients [1,2,3]. COPD is characterized by a steadily progressive course, it has heterogeneous clinical manifestations and it is often associated with a wide range of comorbid diseases, among which cardiovascular diseases take the key position [4]. The inflammation, which is due to prolonged exposure to smoking, is the basis of the development and progression of the disease [4,5]. Despite this, there are relatively few research works describing the triggering events and inadequate regulatory mechanisms, corresponding to them, as well as the subsequent cellular and molecular processes that lead to tissue damage and remodeling.

Inflammation in COPD is characterized by the participation of many cells and humoral factors and is believed to have local and systemic components that can be the basis for the development of comorbid diseases. Comorbidity is a part of the extrapulmonary clinical heterogeneity of COPD and in many respects it determines the nature of the course and prognosis.

Macrophages—key participants in the innate link of the immune system, as indicated by a significant increase in their number in the lungs, take an important place in the pathogenesis of COPD [6,7,8]. It is impossible not to note the role of lipid metabolism disorders besides the innate immune system, to the involvement of which in the development of bronchial inflammation many works are devoted. Macrophages are actively involved in the processes under consideration, and immunometabolic investigations that have been developed in recent years show the importance of lipid metabolism disorders in the basis of dysregulation of the immune response [9]. Cross-links and their disruptions in the regulation of lipid metabolism, the innate immune system, and inflammation may be the key to understanding the pathophysiological bases of COPD.

In this regard, information about the participation of cholesterol and its metabolic pathways in the immune response and inflammation is of considerable clinical interest. The objective of this article is the analysis of the ATP binding cassette transporter A1 (ABCA1 transporter) participation and the function of reverse cholesterol transport in the pathogenesis of COPD. The ABCA1 transporter has a lot of functions, the key to which is participation in the provision of lipid homeostasis of cells. The transport function, the nature of the transported substrates and the cellular localization of the protein have determined its role in many processes and mechanisms underlying the pathogenesis of COPD.

## 2. Disorders of Lipid Metabolism in the Development and Progression of COPD

Despite the active development of medicine, the current understanding of the natural course of COPD is incomplete and often contradictory [10]. It is known that the development and progression of the disease can be very different in different patients, and its onset is not possible to establish. The molecular mechanisms underlying the processes that determine the clinical picture of COPD in different patients or even in one patient at different times, are largely unclear.

Negative dynamics of lung function remains the main criterion for the diagnostics of COPD and a key predictor of the prognosis. The data about an association between a decrease in the airflow parameters and the level of high-density lipoprotein (HDL) cholesterol are interesting. Oelsner E. et al. showed on the basis of a seven-year analysis of a large sample, that higher HDL cholesterol was associated with a higher rate of decrease in FEV1 (*p* < 0.0001) and FEV1/FVC (*p* < 0.0001). The magnitude of indicated effect was similar to the 10-year increase in the pack-years index [11]. Previously the risk of emphysema by 0.4% for every 10 mg/dl increase in HDL cholesterol has already been stated in the MESA LungStudy [12].

The role of high HDL level in the reduction of lung function and in the progression of emphysema has been described previously [12,13,14,15], and it has already been associated with the participation of apolipoprotein M in the previous studies [14,15]. However, there are other data available from the literature indicating a positive association of high HDL levels and pulmonary function. A decrease in HDL in patients with COPD with more severe stages is also noted [15,16,17,18]. It is demonstrated in the recent study that the best predictor of pulmonary function in patients with COPD can be the ratio of lymphocytes to HDL [19]. It is also shown that HDL levels decrease in patients with COPD, who have undergone lung transplantation [20].

In general, these ambiguous data do not correspond to the protective role generally accepted for HDL, according to which they are well known in the pathogenesis of atherosclerosis [21,22]. The results are of more interest if we take into account that COPD and atherosclerosis are often combined and have similar trends of progression.

The presented data allow to suggest that the processes associated with the formation and function of HDL play a significant role in lung function. The differences shown in the data can be related to the clinical heterogeneity of COPD patients, which is associated with individual features of pathophysiological mechanisms and lipid metabolism.

It is necessary to emphasize that the high levels of HDL in the presented studies corresponded to the development of the emphysematous phenotype of COPD. It is known that patients belonging to another, the so-called bronchitic type of COPD, suffer from concomitant cardiovascular diseases, which is associated with an increased predisposition to the development of atherosclerosis in this category of patients. The prevalence of cardiovascular disease and metabolic syndrome in patients with lower body mass index and emphysema is generally relatively low [23,24].

Although the exact mechanisms of the relationship of lung function and the development of emphysema with HDL levels are not clear, various explanations are proposed, for example, the intake of glucocorticosteroids by patients with more severe course of COPD [25].

Thus, the obvious involvement of lipid metabolism in the pathogenesis of COPD, demonstrated by numerous studies, determines the need for a better study of the mechanisms of participation of ABCA1 and reverse cholesterol transport.

In the modern scientific literature there are quite a lot of data describing the biological functions of ABCA1. It belongs to a large family of membrane proteins that transport chemically diverse substrates through the lipid bilayer of cell membranes, accompanied by ATP hydrolysis. In clinical practice mutations in the ABCA1 gene are known as the cause of a rare genetic disease-Tangier disease. The disease is characterized by a significant decrease in HDL level and a high incidence of cardiovascular diseases [26,27,28].

ABCA1 is localized on the plasma membrane of cells and is expressed in many organs and tissues. It participates in the reverse transport of cholesterol, exporting cholesterol and phospholipids from cells to extracellular acceptors [29,30,31]. In addition to the plasma membrane, ABCA1 is also found in the Golgi complex and in lysosomes, which confirms the information about the mobility of the transporter, which can move between the plasma membrane, the Golgi complex, and lysosomes, ensuring the functioning of the lipid transport route [32,33,34,35,36].

ABCA1 expression has complex regulatory pathways that are carried out both at the transcriptional and post-transcriptional levels [37,38,39]. Excess of cholesterol in macrophages leads to the formation of oxysterols that stimulate ABCA1 expression via LXR (liver X receptor) [40,41,42,43]. LXR forms a heterodimer with RXR (retinoid X receptor), and they form a transcription factor together, connecting with specific sites on the ABCA1 gene promoter for ABCA1 expression increase [44,45,46,47].

The participation of the ABCA1 transporter in the formation of HDL has determined its leading role in the pathogenesis of atherosclerosis, which is the subject of researchers’ close attention [48]. However, in recent years there is more and more evidence that the transporter is also involved in the regulation of inflammation [27], which is carried out through various mechanisms, including participation in the immune response and phagocytosis, which increases the interest of clinicians in ABCA1 as the pathogenesis link of other diseases in addition to atherosclerosis [49,50].

The important role of both the transporter itself and lipid homeostasis in lung function in general is indicated by the fact that the expression of ABCA1 in lung tissues takes the second place after the liver [26]. This observation is extremely important, taking into account the great clinical significance of lipids for respiratory function and the influence of oxidative processes to which lipids are exposed during smoking. To the greatest extent, ABCA1 is present in alveolar macrophages, alveolar pneumocytes of types I and II [51,52], which characterizes its involvement in various biological processes [53]. Indeed, the function of the protein is not limited to the simple exchange of cholesterol and understanding all the mechanisms in which the transporter is involved requires serious research.

## 3. Participation of ABCA1 in the Regulation of Inflammation in COPD

It is believed that the ABCA1 transporter can participate in the regulation of inflammation in the lungs. The mechanisms of this involvement are diverse and in many respects are not studied completely. It is assumed that the intracellular accumulation of cholesterol in macrophages acts as a trigger of the cellular inflammatory response [54,55]. Consequently, the ABCA1 transport activity can be anti-inflammatory, through the removal of cholesterol excess [26,56,57,58,59].

There is evidence that cigarette smoke modulates signaling pathways that regulate ABCA1 expression in macrophages [60,61]. The reduced ABCA1 transport activity due to smoking leads to intracellular accumulation of cholesterol or even the formation of so-called “foam cells” [62,63,64]. Research data have shown that ABCA1 expression changes in COPD [60,65]. It was found that in patients with moderate and severe COPD ABCA1 expression in lung tissues was for certain lower than in healthy subjects [60].

The role of ABCA1 in pulmonary inflammation is well demonstrated in mouse models. In ABCA1 knockout mice, compared to the wild type a violation of lipid metabolism is observed, including a significant decrease in plasma the level of cholesterol and phospholipids (by about 70%) due to almost undetectable levels of HDL and ApoA-I [66]. Analyzing these data, it is necessary to note that mice differ from humans in a number of features of HDL metabolism (in mice, unlike humans, protein CETP (cholesterol ester transfer protein) in the blood plasma is absent, it transfers cholesterol esters from HDL to LDL and VLDL containing AрoВ, as a result of which they have naturally low LDL and high HDL levels) [67,68].

In the ABCA1 knockout mice, pulmonary focal lesions were found, including thickening of the interalveolar septa, foam alveolar macrophages and hyperplasia of type II alveolar pneumocytes, the surfactant was characterized by alveolar proteinosis [69,70,71]. With age, the alveolar architecture of these mice was destroyed, and the remaining alveoli were epithelized due to severe hypertrophy and hyperplasia of type II pneumocytes [69,70]. The importance of ABCA1 in normal lung physiology is confirmed by the fact that in mice with ApoA-I knockout increased infiltration by inflammatory cells (especially neutrophils), collagen deposition and airway hyperreactivity, as well as impaired lung vasodilation were observed [69,70,71]. These observations demonstrate the role of ABCA1-mediated reverse lipid transport in inflammatory lung diseases.

### 3.1. Participation of ABCA1 in the Regulation of Inflammation with the Participation of TLR4

The lungs contact constantly with a huge number of pollutants and pathogenic microorganisms through the inhaled air, and therefore they are populated with immune cells densely. As the first line of defense for the lungs, the innate immune system is thought to rely on a large family of PRR (pattern-recognition receptors) for detection of standard molecular structures (patterns) specific to large groups of pathogens, including viruses, bacteria, fungi, parasites, and protozoa. An important role in the initiation of inflammation in COPD is played by Toll-like receptors (TLR) of macrophages, which are a family of transmembrane receptors that are expressed by many cell types, including epithelial cells, endothelial cells, monocytes, macrophages, dendritic cells, and T- and B-lymphocytes [72,73].

The most studied Toll-like receptor TLR4 recognizes lipopolysaccharides of the cell wall of Gram-negative bacteria (LPS) and is localized both on the plasma membrane and in endosomes. When LPS is being recognized, conformational changes in TLR4 receptors lead to the recruitment of its intracellular toll-interleukin 1 receptor (TIR) domain (TIR-domains) containing molecules-adapters, realizing MyD88-dependent and MyD88-independent pathways.

Signaling via TLR4 is of importance in COPD [74,75,76]. In addition to inflammation, it is involved in other processes, such as angiogenesis [77]. TLR4 in the lungs can be activated by either LPS or exogenous oxidants [78] and, consequently, modulate inflammatory responses. It is known that the components of cigarette smoke are also able to activate TLR4, which is important, taking into account its role in the etiology of COPD [79,80].

The evidence that saturated fatty acids can also activate TLR4 is of considerable interest [81,82]. Moreover, unlike saturated fatty acids, unsaturated ones do not have such an effect [82]. These data support clinical observations in which overweight and obese people showed increased TLR4 expression on peripheral blood mononuclear cells and in adipose tissue compared to low body weight, and TLR4 expression levels increased significantly with increase of body mass index [83,84]. Interestingly, that polyunsaturated fatty acids can destabilize ABCA1, disrupt the reverse transport of cholesterol and HDL formation [85,86,87,88,89]. This information is relevant when analyzing the comorbid relationship between COPD and atherosclerosis. There is an assumption that the prevalence of cardiovascular diseases (in particular, coronary artery disease (CAD) and peripheral artery disease (PAD)) may be higher in individuals with a higher body mass index (BMI) and, as it has been noted previously, predominantly bronchitic form of COPD [23,24,90]. At the same time, a low BMI increases the risk of mortality in patients with COPD [91,92], and overweight and obesity are positive predictors of long-term survival [93,94]. This phenomenon got the name “the obesity paradox” [95,96,97]. Its mechanisms are not completely clear, but participation of cytokines, contributing to the development of cachexia, such as TNF (Tumor Necrosis Factor) and IL-6 (Interleukin-6), is assumed [98,99]. TNF is a proinflammatory cytokine that is highly expressed in COPD patients [100]. It is known that TNF activates a number of signaling pathways that induce changes in ABCA1 expression [100], including nuclear factor-kB (NF-kB), sterol regulatory element binding protein 2 (SREBP-2), and janus kinase 2/signal transducer and activator of transcription 3 (JAK2/STAT3) [101,102,103]. However, currently there is contradictory information about the TNF role in the expression of ABCA1 in macrophages and reverse cholesterol transport. In foamy cells derived from THP-1 macrophages, TNF suppresses ABCA1 expression via the NF-kB-dependent pathway [104], while in mouse peritoneal macrophages it induces ABCA1 expression [105].

The results of investigations show that the ABCA1 expression in macrophages can play a physiologically significant role, also through the removal of LPS from macrophages, which helps to restore a normal immune response.

Although TLRs have developed evolutionarily for detection of exogenous pathogens, providing innate immune responses, it is becoming apparent that TLR4 activation is also modulated by endogenous molecules, including lipids.

Recent data suggest that TLR 4, when activated, is localized in the so-called “lipid rafts” of plasma membranes, which can regulate its activity [106]. The plasma membrane is heterogeneous in its structure and functions. The structure of the plasma membrane that separates cells from their environment is the subject of numerous studies. According to current data, the plasma membrane is represented by a lipid bilayer containing various types of lipids distributed asymmetrically between two bilayer sheets in which proteins are embedded. In addition, plasma membranes are assumed to contain so-called lipid rafts-dynamic signaling platforms, which are areas of the plasma membrane enriched with glycosphingolipids, sphingomyelin, cholesterol, glycophosphatidylinositol anchor proteins and signaling proteins [104,107,108,109].

Cholesterol is the main component of lipid rafts, accounting for about 50% of the lipids present in these domains. Cholesterol has many functions, including participation in maintaining the spatial structure of the plasma membrane. In this case, most cholesterol molecules are located by their hydroxyl groups close to the glycerol region of the framework of the lipid bilayer, and their hydrophobic rings are located in the hydrophobic core of the bilayer. Changes in the cholesterol content in the plasma membrane affect its structure and function. Due to the influence on the biophysical properties of the membrane, as well as through the direct interaction of the sterol with specific protein sites, cholesterol can participate in the regulation of the function of transmembrane proteins [110]. It has been shown that proteins that interact with cholesterol or bind it may contain characteristic amino acid sequences that play a definite role in this interaction [106].

**Figure 1:**
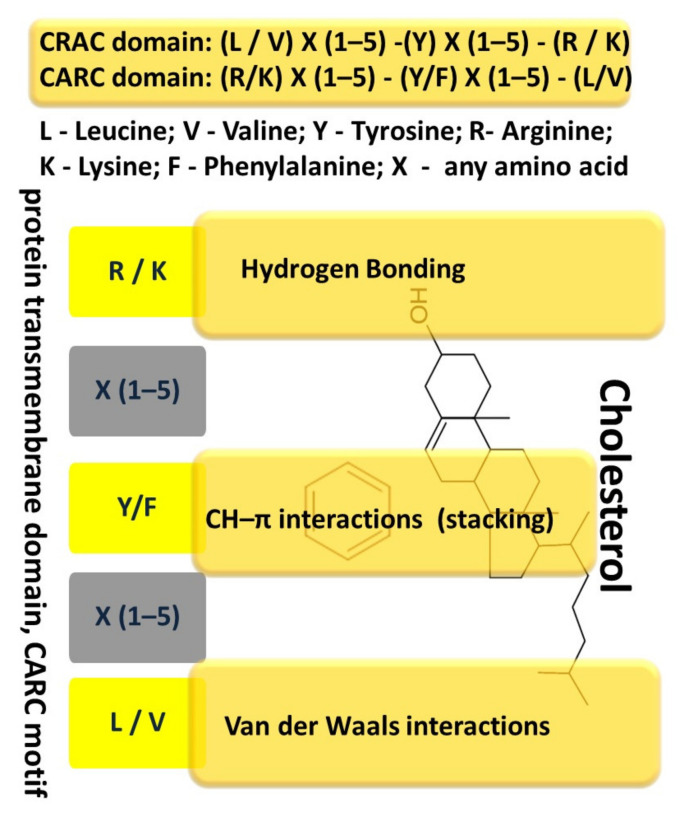
Cholesterol-binding domain (CRAC and CARC domains).

One such known sequence, the amino acid cholesterol-binding domain (CRAC, Cholesterol Recognition/interaction Amino acid Consensus sequence), has been identified in proteins that interact with cholesterol or are regulated by it [102,111,112,113]. The amino acid sequence, referred to as CRAC, is defined by the following set of amino acids: (L/V)–X_(1–5)_–(Y)–X_(1–5)_–(R/K) [112,112], the CARC motive, has similar properties in binding to transmembrane proteins and has an inverse sequence of amino acids: (R/K)–X_(1–5)_−(Y/F)–X_(1-5)_−(L/V) (with X = any amino acid), and tyrosine can be replaced by phenylalanine [112] (Figure 1).

Taking into account, that the probability of the presence of such a domain is very high for many proteins, it is believed that the very presence of CRAC does not indicate the occurrence of specific cholesterol–protein interactions [112,114] and experimental data are necessary for their confirmation [115]. It is also assumed that the CRAC sequence contributes to the localization of membrane proteins in lipid rafts to a certain extent [116]. The presence of several CRAC or CARC sequences, in the transmembrane region or near it, may indicate a possible participation of cholesterol in the accomplishment of protein function.

Analysis of the TLR4 receptor structure allows to reveal the presence of both CRAC and CARC sequences near the transmembrane domain, which can provide a link between cholesterol and the regulation of signal transduction of the receptor [106]. Interestingly, that the CARC-CRAC-CARC domains in TLR4 are located close to the membrane, in front of the TIR domain. This may indicate that this intracellular region of TLR4 binds cholesterol specifically [106].

The interaction of transmembrane proteins, including TLR4, with cholesterol is possible due to its structure. Cholesterol is a polycyclic amphipathic molecule derived from sterane. The cholesterol molecule has a polar and apolar parts. The polar part is represented by a hydroxyl group, which allows to establish hydrogen bonds. The apolar part has an asymmetric structure, including a flat α surface and a β surface with aliphatic groups (two methyl groups and a terminal isooctyl chain). Sphingolipids usually interact with the α-surface of cholesterol, and transmembrane domains of proteins interact with the β-face [112,117]. It is believed that the side chains of branched amino acids, such as valine or leucine, can “permeate” these aliphatic groups and therefore they are particularly suitable for association with the β surface of cholesterol [112] through numerous van der Waals contacts between these residues and cholesterol. The interaction between the aromatic amino acid and cholesterol occurs in the apolar region of the membrane, far from the hydroxyl group of cholesterol, and the interaction with cholesterol is mediated almost exclusively by the CH-π-stacking binding between the aromatic ring of the amino acid (either tyrosine or phenylalanine) and one of the sterane rings of cholesterol [110,117] (Figure 1).

Thus, ABCA1, by changing the cholesterol content in the plasma membranes of macrophages and ensuring the stability of lipid rafts, can regulate the activity of TLR4 [60], which can occur in COPD. In its turn, TLR4 activation inhibits ABCA1 expression, which reduces the outflow of cholesterol from macrophages [118,119]. The activity of IRAK1 (IL-1R-associated kinase 1) plays a key role in this process [120].

Studies of the biological role of ABCA1 have determined that in addition to lipid export, it can mediate intracellular cholesterol transport and allow the movement of lipids between the inner and outer sheets of the plasma membrane [28,121]. These findings have improved our understanding of the role of the ABCA1 transporter in inflammation through the regulation of phosphatidylinositol 4,5-bisphosphate [PI(4,5)P2] in plasma membranes. PI(4,5)P2 is the main cellular type of PIP and it is mainly localized in the inner sheet of the plasma membrane, where it is involved in many cellular processes, such as endocytosis, exocytosis, protein transport, and receptor-mediated signal transduction [122,123,124,125,126]. In the plasma membrane, it is concentrated mainly in lipid rafts. It has been found out that ABCA1 participates in the exchange of PI (4,5)P2, redistributing it from the inner to the outer sheet of the plasma membrane [123,125]. It is believed that PI (4,5) P2, moved to the outer side of the plasma membrane, ensures the binding of apoA-I and the outflow of lipids during the formation of HDL. In addition to it, ABCA1 exports PI (4,5)P2 to nascent HDL, reducing its amount in the plasma membrane [122]. Accordingly, a decrease in the functional activity of ABCA1 may increase the amount of PI (4,5)P2 in the inner sheet of the plasma membrane.

**Figure 2:**
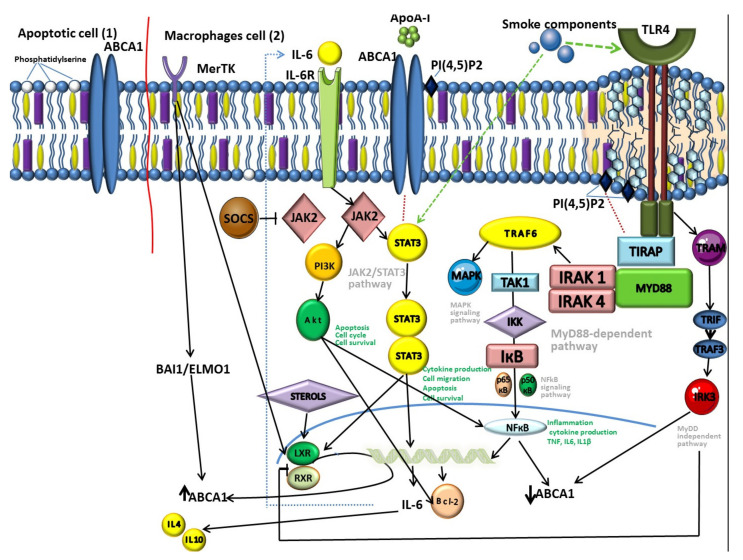
Schematic picture of the ATP binding cassette transporter A1 (ABCA1) participation in the mechanisms of inflammation in macrophages in chronic obstructive pulmonary disease (COPD) (1) and apoptosis (2).

Recent data have shown that PI (4,5)P2, localized in the outer sheet of the plasma membrane, participates in the regulation of cell adhesion and cell motility [126]. One of the most important biological functions of PI(4,5)P2 is related to the fact that it is necessary for the functioning of the sorting adapter TIRAP, a member of the MyD88-dependent TLR4 pathway (Figure 2) [127,128,129]. The MyD88-dependent pathway is regulated by two adapter-associated proteins: Myeloid differentiation primary response gene (88) (MyD88) and toll-interleukin 1 receptor (TIR) domain containing adapter protein (TIRAP) [130].

The MyD88 protein is a key link in the inflammatory signaling pathways of Toll-like receptors (TLR) and interleukin-1 (IL-1) receptors [131]. MyD88 forms a protein complex with kinases of the interleukin-1 receptor-associated kinase (IRAK) family, called the Middosome. MyD88 has a limited ability to interact directly with TLR. This requires an intermediate protein that binds activated TLRs to MyD88. Most plasma membrane-localized TLRs, including TLR4, use the TIRAP sorting adapter to recruit MyD88. The ability of TIRAP to function as a sorting adapter depends on its N-terminal motive rich in positively charged lysine residues [106,128], which interacts with PI (4,5) P2 and other lipids (phosphatidylserine (PS)) localized on the plasma membrane.

Thus, ABCA1, through the transport of PI (4,5)P2, can participate in the regulation of signal transmission along the MyD88 dependent TLR4 pathway [128,132,133].

### 3.2. ABCA1 Cross-Links and JAK2/STAT3 Pathways

It is interesting to learn about another mechanism associated with inflammation, in which the transporter is involved, and which is implemented in parallel with the reverse transport of cholesterol. It has been found out that the interaction of ABCA1 and ApoA-I increases phosphorylation and activates JAK2, which, in its turn, increases the binding activity of ApoA-I and ABCA1, which is responsible for the export of lipids [57,134,135]. At the same time, JAK2 increases the transport activity of ABCA1 [136,137], which is known to have an anti-inflammatory effect.

JAK2, activated by the interaction of ABCA1 and ApoA-I, activates STAT3 additionally [57,138], which is independent of the lipid transport function of ABCA1 [37]. ABCA1 contains two potential docking units with STAT3, necessary for phosphorylation of the latter by ApoA-I/ABCA1/JAK2 [136]. It is considered that the transcription factor STAT3 mediates IL-6 signaling pathways [57,138] and performs an anti-inflammatory function in macrophages [138,139]. This fact allows to suggest the functioning of ABCA1 as a direct anti-inflammatory receptor due to the activation of JAK2/STAT3 [37,138].

The JAK2/STAT3 pathway can also exhibit a proinflammatory effect [57,138,139,140,141]. Such multidirectional participation highlights the complexity of parallel processes and their insufficient investigation. STAT3 regulates a number of fundamental cellular processes, including inflammation, proliferation, differentiation, and cell migration [142]. STAT3 can also regulate apoptosis by inducing the expression of the apoptosis inhibitor Bcl-2 (B-cell lymphoma 2) [143,144].

The JAK2/STAT3 pathway is involved in the regulation of airway inflammation in COPD (Figure 2) [144]. This agrees well with the fact that cigarette smoke, the main etiological factor of COPD, can activate STAT3 in the lungs [44,144,145]. In patients with COPD the STAT3 expression in the lung tissue is increased significantly [146,147]. Moreover, activation levels correlate with the degree of bronchial inflammation, but not with air flow obstruction [148]. This may be explained by the function of STAT3 in the regulation of inflammation, protease production and apoptosis [149,150,151], underlying the pathogenesis of COPD [152,153].

Taking into account the fact that many of the cytokines considered to be the key participants of the persistent inflammation observed in COPD implement their action through JAK/STAT or are produced as a result of its activation [153,154], the interest of researchers in this pathway has increased significantly in recent years. It is noteworthy that ABCA1 mutations, violating the ABCA1/STAT-3 complex did not affect the lipid outflow of ABCA1, but blocked the ability of ABCA1 to suppress cytokine secretion in response to LPS [155].

The data, received as a result of experiments, indicate that the cholesterol load of macrophages associated with ABCA1 inhibition leads to an increase in IL-6 production [118]. In this regard, the increased levels of IL-6 observed in the induced sputum and lung tissue of patients with COPD [156,157] are in good agreement with the data on the reduction of the ABCA1 transport function in macrophages during smoking, as well as with the fact that IL-6 is known to activate STAT3 [158].

IL-6 is well known as a participant in the pathogenesis of many diseases [159], and is often considered as one of the factors of systemic generalization of inflammation and comorbidity of COPD. It is known that IL-6 contributes to the development of emphysema due to activation of STAT3-independent apoptosis [148]. In addition, a correlation between high IL-6 level and mortality of COPD patients has been demonstrated previously [160,161]. At the same time, it should be noted that the function of IL-6 is complex and not unambiguous. It is believed that IL-6 can control inflammatory processes associated with the involvement of adaptive and innate immunity. For example, it can attract myeloid cells to areas of inflammation [162,163]. The experimental results indicate that IL-6 can reduce the proinflammatory response of human macrophages due to induction of anti-inflammatory IL-4 and IL-10 and secretion decrease of the proinflammatory cytokine Il-1β [118]. IL-10 induced by IL-6 may be involved in activation support of STAT3 in macrophages with the help of the specific receptor IL-10R [118,164].

It is also interesting that IL-6 induces ABCA1 expression and enhances the transporter-mediated outflow of cholesterol from human macrophages to apoA-I with the participation of the JAK-2/STAT3 pathway [118,165]. Thus, lipid-loaded macrophages, producing a significant amount of IL-6 promote the induction of ABCA1 gene expression, which leads to an increase in ABCA1-mediated cholesterol outflow through activation of the Jak-2/STAT3 pathway, thereby reducing the formation of foam cells and the accumulation of free cholesterol, respectively.

It has been found out that not only IL-6, but also a number of other cytokines can affect the expression of ABCA1. They can inhibit it as, for example, interferon (IFN) -γ, IL-1β or Platelet-derived growth factor (PDGF), or enhance it as anti-inflammatory cytokines such as IL-10 and TGF-β1 [37].

These and other data suggest that in cholesterol-loaded macrophages Jak2/STAT3 may represent a key signaling pathway for weakening both the accumulation of cellular lipids and the proinflammatory response [57,118,166]. Such anti-inflammatory effect corresponds to the previously described mechanism, in which the interaction of apoAI with ABCA1 activates the Jak-2/STAT3 pathway and participates in the establishment of an anti-inflammatory response in human macrophages [138,166].

The presented data correspond to the concept of macrophage heterogeneity in COPD, according to which proinflammatory M1 and “alternatively activated” (anti-inflammatory, reparative) M2 macrophages producing proinflammatory (including TNF, IL-1ß, IL-6) and anti-inflammatory (e.g., IL-10) cytokines, respectively, are found in the focus of persistent inflammation at the same time [6,167].

However, in COPD, there are obviously violations of the described anti-inflammatory mechanisms, and the implementation of the JAK-2/STAT3 pathway may be one of the links in a complex chain of mechanisms of COPD pathogenesis [144].

### 3.3. Other Mechanisms of ABCA1 Participation in Inflammation

There are other known mechanisms of inflammatory activation of macrophages associated with intracellular cholesterol accumulation. Intracellular cholesterol in crystalline form can participate in the activation of inflammation via Nod-like receptors (NLR) acting as intracellular observation molecules [168,169,170,171]. The ability of various crystalline substances to activate NLRP3 inflammation is well known for many both exogenous substances and endogenous molecules, for example, silicon dioxide and monosodium sulfate, and it has also been described for cholesterol crystals [168,169,170,171]. It should be noted that nowadays the possibilities of activation of NLRP3 inflammation in COPD due to the accumulation of crystalline forms of cholesterol in myeloid cells are not clear, but this is well known by the example of atherosclerosis [168,169,170,171,172]. At the same time, recent studies have shown that NLRP3 is highly expressed in the lungs, which is due to the large number of immune cells characteristic of this organ, and emerging scientific evidence suggests that the activation of NLRP3 inflammasome may be involved in the onset of COPD pathogenesis [173,174,175,176].

Infectious exacerbations of COPD are an important characteristic of the disease and largely determine the rates of progression and prognosis. It has been found out that bacterial colonization of the bronchi makes a contribution to the progression of the disease. Bacterial colonization of the bronchi caused by a defect in phagocytosis in COPD [177]. *Streptococcus pneumoniae*, *Haemophilus influenzae*, *Moraxella catarrhalis* and *Pseudomonas aeruginosa* are most often found in the lower respiratory tract in COPD [178,179]. Bacterial colonization leads to dysregulation of the immune response, also with the participation of TLR4.

Information about the ability of some bacteria to influence ABCA1-mediated lipid transport in lung epithelial cells is interesting. In particular, it has been shown that Ps. aeruginosa can impair lung function through the induction of ABCA1-mediated export of the surfactant phosphatidylcholine (PtdCho) in the alveolar epithelium of mice. The regulation of the transporter expression is probably carried out via the PPARa (peroxisome-proliferator-activated receptor-a)/RXR pathway [135]. Previously it has been found out that the effect of LPS on macrophages leads to a rapid dose-dependent increase in the expression of ABCA1 mRNA [180]. It has also been shown that infection *Chlamydia pneumoniae* infection reduces ABCA1 expression in A549 lung epithelial carcinoma cells [66].

In general, violations of ABCA1-mediated lipid transport due to bacterial colonization of the bronchi are of considerable clinical and research interest for assessment its role in infectious exacerbations of COPD.

## 4. Participation of ABCA1 in Phagocytosis and Apoptosis

Due to chronic inflammation of the respiratory tract in COPD there is a significant increase in the number of cells undergoing apoptosis [181]. Apoptosis is the most important mechanism for ensuring of cell self-renewal and plays an important role in responses to damage or infection by controlling the number of cells involved in the process of inflammation [182,183]. The removal of apoptotic cells is mainly carried out by macrophages in the process of efferocytosis [181].

It is known that efferocytosis is impaired in patients with COPD, but the mechanisms of these disorders are not clear nowadays [184,185]. An accumulation of apoptotic epithelial, endothelial, and immune cells in the lungs is noted in these patients [186,187,188]. In addition, the induction of structural apoptosis of airway cells may be the cause of the development of emphysematous changes [189].

Effective removal of cells that have undergone apoptosis from tissues requires their specific recognition either by neighboring cells or by specialized phagocytes [182,190]. Although cells undergoing apoptosis retain the integrity of the plasma membrane, the resulting changes in the composition of membrane lipids, carbohydrates and proteins provide the necessary molecular signals, marking them for recognition by other cells. One of the signs of apoptosis is the translocation of phosphatidylserine (PtdSer) from the inner sheet of the plasma membrane to the outer sheet [182,183]. It has been shown that this process is connected with the ABCA1 transporter function [191,192]. The involvement of the transporter in phagocytosis is well known from the animal models, that we have analyzed before [193].

It is believed that the localization of phosphatidylserine in the outer sheet of the plasma membrane of apoptotic cells is an almost universal signal for recognition by phagocytes [182,194,195]. This process occurs due to binding to various receptors on the cell surface of phagocytes, for example, merthyrosine kinase (MerTK) [44,196,197]. An increased expression of MerTK on the macrophages of the respiratory tract is detected in smokers [198].

Studies have shown that during MerTK-regulated efferocytosis in lung tissue, the LXR pathway is activated, resulting in increased expression of ABCA1 (Figure 2) [181,192,199]. The independent of LXR pathway of ABCA1 activation in phagocytic macrophages, such as BAI1/ELMO1/Rac, is known [200]. This pathway includes receptor brain-specific angiogenesis inhibitor 1 (BAI1), which recognizes phosphatidylserine on apoptotic cells. BAI1 is also identified as a pattern recognition receptor (PRR), which detects LPS and mediates phagocytosis of Gram-negative bacteria by macrophages [201,202]. Despite this, the role of BAI1 in COPD has not been known yet.

The increase of ABCA1-mediated reverse transport of cholesterol from macrophages during phagocytosis is physiologically explicable. As phagocytes absorb objects, rich in cholesterol, ABCA1 provides protection of cells from its overload [104]. Although ABCA1 does not participate directly in the phagocytosis of apoptotic cells, it can help phagocytes recover [104,191]. In addition, ABCA1 induction provides protection of macrophages from oxidative stress caused by the uptake of oxidized lipids [104].

## 5. Participation of ABCA1 in the Development of COPD Phenotypes

The clinical heterogeneity of COPD is an important characteristic of the disease. The mechanism explaining why different patients with the same risk factor have an emphysematous or bronchitic phenotype is largely unknown. In accordance with the vascular hypothesis, it is assumed that the development of emphysema in COPD may include the progressive loss of endothelial and epithelial cells in the process of apoptosis [203,204,205,206,207]. The results of the studies indicate that vascular endothelial growth factor VEGFA is involved in this process [208,209]. Its role in the pathogenesis of COPD is diverse. The activation of the VEGFA pathway explains the hyperproduction of mucus in the bronchitis phenotype, since VEGFA was originally described as a factor, increasing vascular permeability [210]. Inhibition of the VEGFA pathway, on the contrary, contributes to the disruption of endothelial cell renewal, avascularization of the alveolar septa and their subsequent destruction [209,211]. Experiments on animals have confirmed that blockade of the VEGFA signaling pathway with a VEGFR inhibitor led to apoptosis of endothelial cells in the lungs and morphological changes characteristic of emphysema [212].

**Figure 3:**
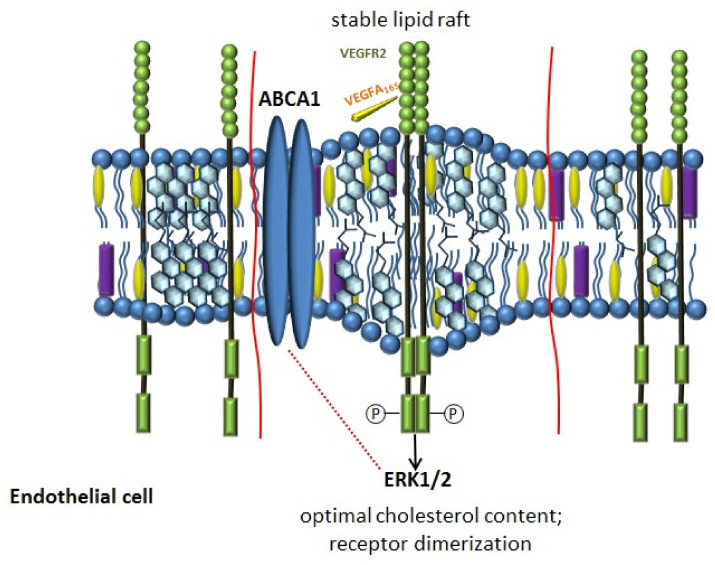
Schematic picture of the ABCA1 transporter participation in angiogenesis through regulation of the vascular endothelial growth factor (VEGFA) pathway in endothelial cells in COPD.

It is believed that a decrease, as well as an increase in the amount of cholesterol in the plasma membrane, affects the structure of lipid rafts and disrupts the signaling of VEGFR2, the main angiogenic receptor on the cell membrane [213]. The restoration of the normal cholesterol content in the lipid rafts of endothelial cells stabilizes the dimeric state of VEGFR2 and angiogenesis [213]. Thus, ABCA1, providing the stability of lipid rafts through the regulation of cholesterol content in the plasma membrane, can participate in the signal transduction of VEGFR2 (Figure 3) [214]. The results of the studies confirmed that the activation of LXR can disrupt angiogenesis, which is associated with their effect on the homeostasis of endothelial cholesterol [214,215,216].

VEGFA, binding to VEGFR2 triggers its autophosphorylation, activating various intracellular pathways, including ERK1/2 [216]. ERK1/2 are involved in many cellular processes, such as embryogenesis, differentiation, proliferation, and cell death [217,218]. It has been shown that inhibition of ERK1/2 affects both the expression and the activity of ABCA1 [219,220,221]. In macrophages inhibition of ERK1/2 induces expression of ABCA1 [219] via LXR, which significantly increases cholesterol export. In general, the available literature data allow to suggest that ERK1/2 activity may play an important role in the cholesterol metabolism of macrophages.

It has been shown that HDL can also affect angiogenesis, demonstrating both pro- and antiangiogenic actions, which are implemented in several ways, including through VEGFA and sphingosine-1-phosphate (S1P) [213,222,223,224,225]. Moreover, ABCA1 may be involved in the regulation of these pathways [226]. In vitro, under hypoxic conditions, reconstituted HDL (rHDL) enhanced VEGFR2 activation and enhanced phosphorylation of the downstream angiogenesis signaling proteins ERK1/2 and p38 MAPK [222]. Thus, promoting angiogenesis, induced by ischemia, HDL can suppress angiogenesis induced by inflammation [227,228,229]. The antiangiogenic action can be realized by suppression of NF-kB and activation of macrophages [228].

Thus, the ABCA1 transporter may be involved in the pathogenesis of COPD phenotypes. The development of emphysema involves several mechanisms, many of which have not been identified yet. ABCA1 is involved in angiogenesis, apoptosis, and inflammation, which indicates its significant role not only as a simple lipid transporter, but as a regulator of many important biological processes.

## 6. Conclusions

Thus, the mechanisms of COPD initiation, development and progression are complex and involve many different pathways. At the same time, the importance of lipid metabolism disorders in these processes does not raise any doubts.

The performed analysis of the data showed that ABCA1 can take part in various processes that are disrupted in COPD. These conclusions are supported by the fact that tobacco smoke, the main etiological factor of COPD, can disrupt the expression and function of ABCA1.

ABCA1 in COPD, due to a violation of its lipid transport function, does not provide adequate reverse transport of cholesterol in macrophages, which may cause inflammatory activation of these cells. The mechanisms through which the transporter is involved in the activation of inflammation are different. The analysis of the data from available studies has shown that one of these key pathways is the participation of the transporter in the activation of the TLR4 receptor signaling pathway, which is well known in the pathogenesis of COPD. Activation of both TLR4 itself and its signaling pathway links is possible through participation in the stabilization of lipid rafts, the accumulation of excess cholesterol in cells, and participation in ensuring the functioning of the descending links of the signaling pathway.

It is interesting to learn about the presence of crosstalk between ABCA1 and the JAK2/STAT3 pathway, which takes an active part in the pathogenesis of COPD, demonstrating both anti- and proinflammatory properties, which shows the complexity of simultaneous processes. The associations of ABCA1 with cytokines such as IL-6, IL-1β, which are involved actively in the pathogenesis of COPD, in addition enhance the significance of the transporter and disorders of its functioning.

Taking into account the fact that part of the pathogenesis of COPD is an increase in bacterial colonization of the bronchi, as well as the presence of infectious exacerbations of the disease, the possible participation of bacteria in the regulation of ABCA1 activity opens up new prospects for studying these relationships. The crosstalk of ABCA1 and TLR4, which is also involved in bacterial colonization of the bronchi, reinforces this interest.

Since disorders of phagocytosis and apoptosis are an important link in the development of COPD, data on the participation of ABCA1 in these processes are significant. The function of the protein in this process is related to the fact that the reverse transport of cholesterol, mediated by ABCA1, provides protection of cells from cholesterol overload during phagocytosis. In addition, ABCA1 participates in the exposure of phospholipid ligands on the surface of apoptotic cells.

It is known that COPD is a clinically heterogeneous disease. This heterogeneity includes both pulmonary and extrapulmonary components, such as cardiovascular comorbidity. The involvement of ABCA1 in the pathogenesis of atherosclerosis, through the function of reverse cholesterol transport, as well as participation in the development of lung emphysema through the regulation of angiogenesis, indicate an important contribution of the transporter to various mechanisms of COPD development.

Thus, the closely intertwined disorders of ABCA1-mediated cellular lipid export, homeostasis of membrane lipid rafts and inflammatory activation of macrophages make a significant contribution to the pathogenesis of COPD [230].

The performed analysis showed that ABCA1 and reverse cholesterol transport are involved in many links of the pathogenesis of COPD and, accordingly, take part in determination of the character of the natural course of the disease, including a decrease in lung function, infectious exacerbations, pulmonary and extrapulmonary clinical heterogeneity.
